# Longitudinal Effects of Bumetanide on Neuro-Cognitive Functioning in Drug-Resistant Epilepsy

**DOI:** 10.3389/fneur.2019.00483

**Published:** 2019-05-08

**Authors:** Zeinab Gharaylou, Lida Shafaghi, Mohammad Ali Oghabian, Ali Yoonessi, Abbas Tafakhori, Esmaeil Shahsavand Ananloo, Mahmoudreza Hadjighassem

**Affiliations:** ^1^Department of Neuroscience and Addiction Studies, School of Advanced Technologies in Medicine, Tehran University of Medical Sciences, Tehran, Iran; ^2^Neuroimaging and Analysis Group, Imam Khomeini Hospital, Tehran University of Medical Sciences, Tehran, Iran; ^3^Imam Khomeini Hospital, Iranian Center of Neurological Research, Tehran University of Medical Sciences, Tehran, Iran; ^4^Roozbeh Hospital, Tehran University of Medical Sciences, Tehran, Iran; ^5^Brain and Spinal Cord Injury Research Center, Neuroscience Institute, Tehran University of Medical Sciences, Tehran, Iran

**Keywords:** bumetanide, drug-resistant epilepsy, GABA, TRACULA, cognitive function

## Abstract

Antiepileptic drugs (AEDs) have repeatedly shown inconsistent and almost contradictory effects on the neurocognitive system, from substantial impairments in processing speed to the noticeable improvement in working memory and executive functioning. Previous studies have provided a novel insight into the cognitive improvement by bumetanide as a potential antiepileptic drug. Through the current investigation, we evaluated the longitudinal effects of bumetanide, an NKCC1 co-transporter antagonist, on the brain microstructural organization as a probable underlying component for cognitive performance. Microstructure assessment was completed using SPM for the whole brain assay and Freesurfer/TRACULA for the automatic probabilistic tractography analysis. Primary cognitive operations including selective attention and processing speed, working memory capacity and spatial memory were evaluated in 12 patients with a confirmed diagnosis of refractory epilepsy. Participants treated with bumetanide (2 mg/ day) in two divided doses as an adjuvant therapy to their regular AEDs for 6 months, which followed by the re-assessment of their cognitive functions and microstructural organizations. Seizure frequency reduced in eight patients which accompanied by white matter reconstruction; fractional anisotropy (FA) increased in the cingulum-cingulate gyrus (CCG), anterior thalamic radiation (ATR), and temporal part of the superior longitudinal fasciculus (SLFt) in correlation with the clinical response. The voxel-based analysis in responder patients revealed increased FA in the left hippocampus, right cerebellum, and right medial temporal lobe, while mean diffusivity (MD) values reduced in the right occipital lobe and cerebellum. Microstructural changes in SLFt and ATR accompanied by a reduction in the error rate in the spatial memory test. These primary results have provided preliminary evidence for the effect of bumetanide on cognitive functioning through microstructural changes in patients with drug-resistant epilepsy.

## Introduction

Impairment in diverse cognitive domains is a frequently occurring component in epileptic syndromes either due to the natural course of the disease or the antiepileptic drug (AED) related adverse effects. Various forms of epilepsies cause neurocognitive abnormalities such as substantial memory disturbances (1–3). The specific drug-induced dysfunctions which are irreversible in some circumstances could deleteriously affect medication tolerability, behavioral performance, and psychosocial functioning within a long-term scope (4–6). Even with these cognitive sequels, a number of antiepileptic drugs have been able to improve executive functions in some ways (7). Consequently, the assessment of the AEDs associated cognition-reshaping impact would make a substantial contribution to medicinal progression in these interacting fields (7). Prior investigations have broadly signified the role of gamma-aminobutyric acid (GABA) system as the main inhibitory agency in mediating epileptogenesis/anti-epileptogenesis processes and shaping the fundamental cognitive constructs like memory consolidation (8, 9). The overexpression of NKCC1, a chloride concentration regulating transporter, and resulting abnormal alterations in GABA functional polarity have been reported in several neuropsychological disorders such as epilepsy, tuberous sclerosis complex (TSC), Down syndrome, Huntington disease and autism spectrum disorders (10–12). Bumetanide is a sulfonamide-derived loop diuretic, which powerfully blocks NKCC1 co-transporter (12). We have already reported that bumetanide could be a potent and safe adjuvant therapeutic agent in seizure controlling protocols (13, 14). Despite the low concentration of bumetanide in the brain environment, studies on Huntington and Down's model revealed that restoring the inhibitory function of GABA -as the major probable mechanism of bumetanide- might assist in memory improvement through inducing modifications in synaptic plasticity patterns (15–17). In addition, bumetanide improved social stimuli processing in autism (18, 19) and made the behavioral domain indices in TSC more efficient (20). Although the earlier studies provided considerable insights into cognitive recovery by bumetanide, no research has been found that surveyed the neuro-cognitive impacts of bumetanide add-on therapy on patients with pharmaco-resistant epilepsy.

Structural neuroimaging studies have repeatedly reported the association between cognitive deficits and changes in white matter microstructure in epileptic syndromes (3, 21). Structural connectivity and microstructural integrity can be detected by Diffusion Tensor Imaging (DTI) (22). Several cross-sectional evaluations have generally supported widespread white matter diffusion abnormalities in the epileptic brain (23, 24) that measured by fractional anisotropy (FA) and mean diffusivity (MD). Newly developed tractography technique, TRACULA (TRActs Constrained by UnderLying Anatomy), is a powerful whole-brain tractography method which reconstructs 18 major white matter fibers. The principle superiority of TRACULA is its ability to analyze longitudinal tract alterations in a probabilistic way (25, 26); Therefore, through the current study, we aimed to investigate the association between longitudinal effects of bumetanide on the whole brain structure using voxel-based analysis, white matter changes using TRACULA, and their relations to the cognitive performance indices.

## Materials and Methods

Patients with the diagnosis of medically refractory epilepsy entered the study. The criteria were considered according to the International League Against Epilepsy (ILAE), Classification of Epileptic Seizures (27); that defines the resistance state as experiencing at least 1 seizure per month and receiving at least two standard conventional anti-epileptic drugs within a stable regimen Patients excluded if they had any reports of other major neurological or medical comorbidities. Healthy subjects were asked about general medical conditions with emphasizing on head traumas, seizures and neuropsychological disorders regarding themselves or their family members. Less than 6 years of standard education, taking any medication, alcohol or substance use, renal and liver dysfunctions were among the other causes for exclusion. Finally, twelve right-handed, age, gender, and education level matched patients were selected for each group.

Patients monitored 4 months prior to bumetanide administration in order to ensure the steady therapeutic doses and effects. Subsequent to these primary steps, based on observations from our former work (13) bumetanide 2 mg/day was added to the baseline stabilized AEDs. Patients classified as a responder if they had a 50% reduction in the frequency of seizures compared to the baseline (28).

All of the participants or their guardians assigned written informed consent. The study protocol approved by the ethical committee of Tehran University of Medical Sciences and got its' registration code from the Ministry of Health and Medical Education (IRCT201012115368N1). The basic characteristics of the participants are described in Table 1.

**Table 1:** Baseline demographics, AEDs and epilepsy characteristics.

**Age range (number of cases)**	**Seizure type**	**Duration (year)**	**Age of onset (year)**	**MRI finding**	**Baseline AED(s)**	**Response (%)**	**Seizure frequency**
15–25 (6)	^#^CPS	9	6	Normal	LEV (500 mg), VPA(1,000 mg), PHB (50 mg), ZNS (100 mg), VBT(500 mg)	43.6	4/day
	^*^GTCS	15	5	Normal	LEV (500 mg), TPA (50 mg), VPA (500 mg), CLN (1 mg/qhs), PHT(100 mg)	44.4	90/month: During awake and sleeping
	CPS	7	13	Right Parietal FCD	VPA (1,500 mg), CBZ(1,200 mg)	92.8	12/month
	GTCS	22	1	Right Temporal Atrophy	CBZ (600 mg), CLN (1 mg), LTG (600 mg), VPA (600 mg)	60.4	31/month
	CPS	14	10	Normal	VPA (600 mg), TPR (175 mg), CLN(1 mg)	75	10/day
	CPS	13	12	Normal	LEV (1000mg), CBZ (600mg), PHB (50mg), VPA(400mg)	91.6	5/month
26–36 (5)	GTCS	23	3	Normal	LEV (1,000 mg), VPA (1,000mg), TPR (200 mg), PHT (100 mg)	64.1	2/month
	GTCS	28	1	Right Frontal Gliosis	VPA(500 mg), CLN (1 mg,qhs), PHT(100 mg)	60.4	15/month
	GTCS	25	6	Right Parietal FCD	VPA (1,500 mg), TPA (100 mg), CBZ(600 mg)	6.6	4/month: Repeated falling
	CPS	31	2	Right Frontal Gliosis	CBZ (800 mg), PHT (100 mg), Primidone	81.8	1/month
	GTCS	9	24	Right Occipital Gliosis	CBZ (800 mg), LTG(100 mg), ACZ(500 mg)	63.8	8/month
37–47 (1)	CPS	18	24	Normal	VPA (800 mg), CBZ (2,000 mg), CLN(1 mg)	36.6	15/month

AED, Anti-epileptic Drug; CPS, Complex Partial Seizure; GTC, Generalized Tonic Clonic Seizure; FCD, Focal Cortical Dysplasia; LEV, Levetiracetam; VPA, Valproate; PHB, Phenobarbital; ZNS, Zonisamide; VBT, Vigabatrin; TPR, Topiramate; CLN, Clonazepam; PHT, Phenytoin; CBZ, Carbamazepine; LTG, Lamotrigine; ACZ, Acetazolamide.

^*^ and # are based on the new classification of seizure types:

* CPS: Focal impaired awareness seizure.

# GTCS: Focal to bilateral tonic-clonic seizure.

### MRI Acquisition

MRI scans were acquired from the patients in two time-points; once before adding bumetanide to their stable regimen and the second 6 months after receiving bumetanide. The same scanning procedure run one time for healthy controls. We used a 3.0T Siemens Magneto Tim Trio whole-body scanner (Siemens AG, Erlangen, Germany) with a 32-channel head coil. Anatomical images acquired with a high-resolution, T1-weighted MPRAGE (TR = 1,800, TE = 3.44 ms, flip angle = 7°, FOV = 256 mm, matrix = 256 × 256, voxel size = 1 × 1 × 1 mm). One hundred and seventy six contiguous sagittal slices provided whole-brain coverage. The Diffusion Weighted Imaging obtained using a single-shot spin echo EPI sequence. Whole brain diffusion images received b = 700 s/mm^2^ with 30 directions. The repetition time (TR) and echo time (TE) were 13 and 101 ms for this image. Extra brain volumes received four no diffusion weighting (b = 0 s/mm^2^) with opposing phase-encode directions (Anterior-Posterior and opposite). The EPI readout uses the size of 128 × 128 and field of view (FOV) of 256 × 256 mm^2^ and a slice thickness of 2 mm, isotropic voxels of 2 × 2 × 2 mm^3^. Sixty eight slices acquired to cover the whole brain. The total acquisition time for imaging was about 10 min.

### Diffusion Image Processing

#### Whole Brain Voxel-Based Analysis

For each subject, all DWI scans with b = 700 concatenated into a single data set and corrected for the subject head motion. Eddy current and EPI distortions also corrected with required B-matrix adjustments. The tensor model fitted to the corrected data and then mean diffusivity (MD) and fractional anisotropy (FA) calculated from the tensor's eigenvalues (29). Data preprocessing performed using Eddy-FSL (30).

Voxel-based analyses (VBA) were performed using statistical parametric mapping 12 (SPM 12). To create study template, B0 images of control subjects normalized to the Montreal Neurological Institute (MNI) EPI template. Estimated parameters applied to all FA and MD images. Then these images averaged and smoothed with 6 mm full-width at half-maximum (FWHM) smoothing filter. Afterward, native FA and MD images of controls and patients in two-time points normalized to the study templates and smoothed with a 6 mm FWHM. The general linear model (GLM) was set up to evaluate group differences (pre-treatment vs. control). The statistical threshold of SPM [The Family Wise Error (FWE)] used for multiple comparisons correction of the voxel at a *p* < 0.05 level in cluster size ≥20 voxels which were considered as significant. Further on, the mean value of FA and MD in significant areas calculated for longitudinal analysis. Finally, GLM was set up to run paired comparison (*t*-test) in order to assess other brain regional changes between pre- and post-treatment phases.

#### Tracts Statistical Analysis

Longitudinal tractography was performed by TRACULA tool in Freesurfer (31). Automatic reconstruction of major white matter (32) tracts done for control and patient groups (pre- and post-treatment). FA and MD averaged over the entire trajectory of 18 pathways for statistical analysis. The tract of interest consisted of: commissural tracts (Forceps Major and Minor), anterior thalamic radiation (ATR), cingulum-angular (infracallosal) bundle (CAB), cingulum- cingulate (supracallosal) bundle (CCG), corticospinal tract (CST), inferior longitudinal fasciculus (33), superior longitudinal fasciculus [temporal part (SLFt), parietal part (SLFp)], and the uncinate fasciculus (UF) in each hemisphere.

#### Quality Assessment of DTI Data

Since there is a difference in the signal-to-noise ratio (SNR) in diffusion images, we operated our calculations through TRACULA based on the mean of the signal intensity of whole brain images. SNR values averaged for each participant and applied for statistical variability analysis. As the differences in head motion between the study groups could induce false difference diffusion parameters (34). The average of volume-by-volume translation and rotation, the percentage of signal drop-out and also the average of drop-out scores with excessive intensity computed for each subject. Total motion index (TMI) was obtained from the four motion signs and applied as nuisance regressors in group analysis. Total motion index (TMI) calculated for the i-th subject based on the formula that is given by:

(1) $$\begin{array}{r} TMI_{i}=\overset{4}{\underset{j=1}{\sum }}\frac{x_{ij}-M_{j}}{Q_{j}-q_{J}}, \end{array}$$

where j = 1…,4 indexes the four motion measures as mentioned above, x_ij_ is the value of the j-th motion measure of the i-th subject. M_j_, Q_j_, and q_j_ are the median, upper quartile, and lower quartile of the j-th motion measure over all subjects who were included in a group comparison (34).

### Cognitive Assessment

Spatial working memory test was conducted in two steps. Firstly, two pictures were shown in a sequence of 1 s intervals at different locations on the screen and the number of pictures increased up to seven items in a graded state. Secondly, one picture presented in the middle of the screen and participants should recall the correct location of the middle picture. Each participant's mean reaction time and during the task-accuracy were calculated. Moreover, we examined memory by Stroop test and n-back test. The Stroop Color and Word Test (SCWT) consisted of three conditions: neutral, congruent and incongruent. These assessments illustrated the image processing speed and working memory capacity as parts of the executive functions. Tests were computerized using Psychopy software v.1.84.2 and were used through standard operating procedures.

### Outcome Variables

Percentage of changes in seizure frequency was the clinical efficacy index. Seizure frequency calculated according to the following equation:

(Seizure frequency in 1st month+… + Seizure frequency in the *n*th month)/*n*

Patients with at least 50% monthly seizure frequency reduction were considered as responders (Table 1).

### Statistical Analysis

Comparison between the control group and pre-treatment phase of the patient group done with mann-whitney (non-parametric unpaired *t*-test). It should be noted that all these analyses corrected for 18 comparisons (reflecting 18 tracts) using Bonferroni correction and the significance level considered <0.002 (*P* < 0.002). Longitudinal analysis was performed using a linear mixed model in which FA and MD values incorporated in each tract equation as the dependent variables. Being responder or non-responder considered as the between-subject factor while the scan order (Scan-1, Scan-2) and intended hemisphere regarded as within-subject repeated measures. Gender, age, intracranial volume, and TMI considered as the covariates in this analysis. Significant differences in responder group were considered supportive of the study hypothesis. Also, this model determined whether changes in cognitive scores varied in responders, non-responders and over time that bumetanide administered. We calculated FA and MD values of changes between before and after phases of bumetanide administration. Then Spearman correlation analysis applied between them and changes that found through cognitive assessments. *P* < 0.05 was considered as the significance level. SPSS v23.0 (SPSS Inc., Chicago, Illinois) was used for all the performed analyses.

## Results

### Quality Assessment

The SNR comparison showed no significant difference between patients in pre-treatment phase vs. control group; *P* = 0.73, and pre-treatment vs. post-treatment; *P* = 0.64 (mean ± SDs were as follow: control: 3.85 ± 0.29; pre-treatment: 3.80 ± 0.34; post-treatment: 3.85 ± 0.26). Also, there were no significant differences in TMI between groups in two comparison settings (Table 2).

**Table 2:** Head motion parameters for the patients in pretreatment (TP1), post-treatment (TP2), and controls.

**Head motion parameters**	**Control**	**TP1**	**TP1 vs. Control**	**TP2**	**TP1 vs. TP2**
	**Mean** **±** **SD**		***P*-value**	**Mean ± SD**	***P*-value**
Translation (mm)	0.60 ± 0.20	0.51 ± 0.23	0.35	0.51 ± 0.23	0.12
Rotation (degrees)	0.004 ± 0.001	0.004 ± 0.002	0.41	0.004 ± 0.001	0.06
Drop-out percentage	0.00 ± 0.00	0.00 ± 0.00		0.00 ± 0.00	
Drop-out severity	1.00 ± 0.00	1.04 ± 0.00		1.01 ± 0.00	

*Translation, average volume-by-volume translation; Rotation, average volume-by-volume rotation angles; Drop-out percentage, percentage of slices with signal drop-out; and Drop-out severity, signal drop-out severity*.

### Clinical Response to Bumetanide

The clinical response to the bumetanide add-on therapy defined as more than 50% reduction (35) in seizure frequency after the therapeutic course (calculated seizure frequency during first month of assessment compared to the last month of evaluation). Eight patients (67%) from all twelve subjects that completed 6 month experiment fulfilled this criterion and classified as responders. Two patients among the responders became close to the seizure-free state with more than 90% reduction (91.6 and 92.8%) (Table 1).

### Bumetanide Effect on Longitudinal Changes of the Whole Brain

We performed a whole-brain voxel-based analysis using FA and MD maps for the unpaired two-sample *t*-test in order to run the comparison between patients in the pre-treatment phase and controls. In longitudinal assessment mean value of FA and MD that differed significantly between control and patients applied for subsequent analyses after medication. LMM assessment in responder patients revealed increased FA in the left hippocampus, right cerebellum, and right medial temporal lobe, while MD value reduced in the right occipital and cerebellum. These data suggest that reduction in seizure is accompanied by reversion in diffusion parameters. MNI coordinates, cluster size and *Z*-values of finding shown in Table 3.

**Table 3:** Significant changes in DTI parameters after bumetanide administration in regions that found abnormal in pre-treatment vs. control subjects using a correction for multiple comparisons (36) at the cluster level (*p* < 0.05).

**Left/** **Right**	**Location**	**K**	***Z*-score**	**MNI coordinates (X, Y, Z)**			**Response *P*-value**
**FA:**							
Left	Hippocampus	209	6.39	−14	−20	−16	0.01^*^
Left	Post central	59	6.58	−20	−36	−2	–
Left	Cerebellum	20	6.66	8	−48	−30	–
Right	Post central	23	6.47	−48	−10	22	–
Right	Cerebellum	30	6.45	10	−46	−36	−0.002^**^
Right	Temporal	36	6.47	28	−20	−8	0.01^**^
Right	Caudate	207	6.55	10	4	0	–
**MD:**							
Left	Pre-central	1,455	7.25	−24	−18	70	–
Left	Insular	386	7.20	−38	10	−12	–
Left	Parietal	440	6.90	−32	−70	36	–
Left	Frontal	20	6.14	−20	52	−10	–
Right	Frontal	286	6.33	4	0	38	–
Right	Occipital	123	6.55	32	−90	8	0.05^*^
Right	Temporal	136	5.97	66	−18	6	–
Right	Cerebellum	121	5.56	24	−86	−32	0.003^**^

FA, Fractional Anisotropy; MD, Mean Diffusivity.

* P < 0.05;

** *P < 0.01*.

**Figure 1:**
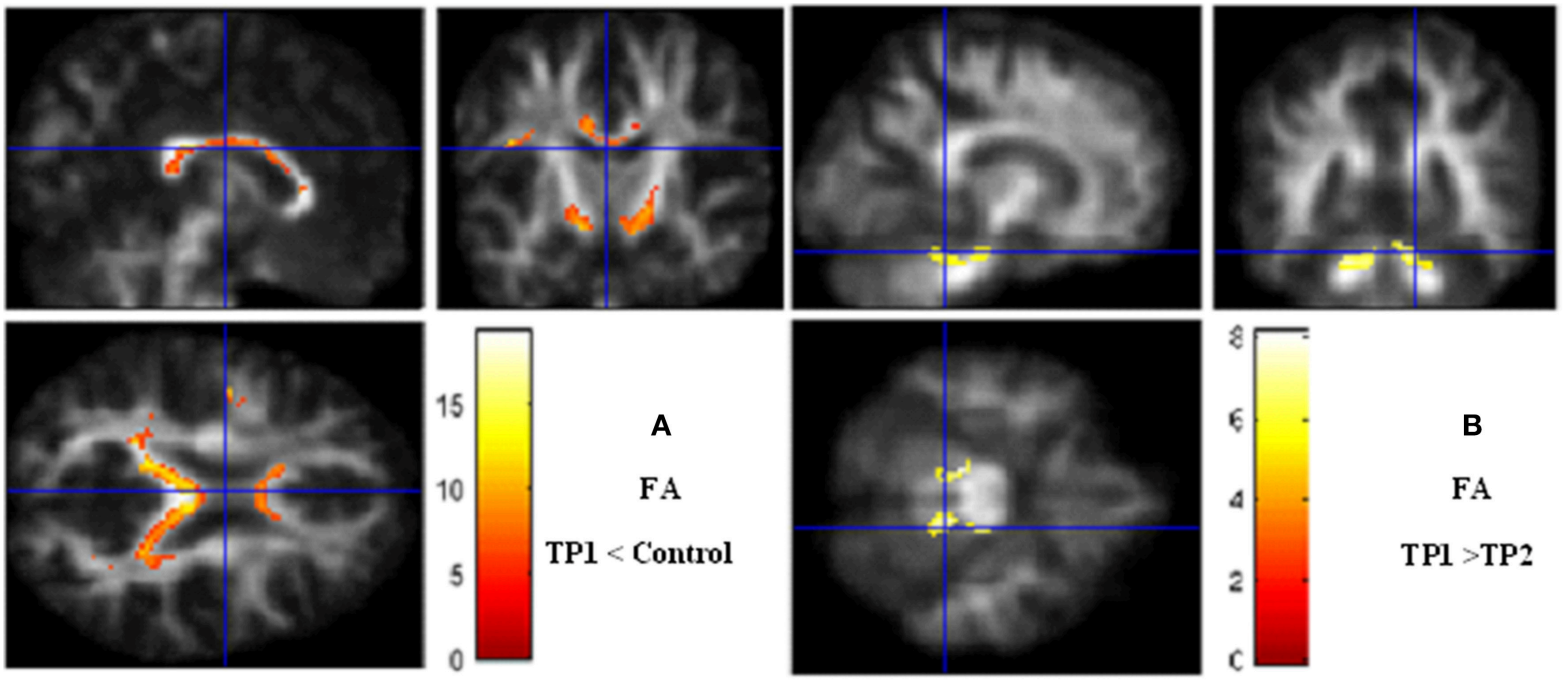
Voxel-based analysis. **(A)** Fractional anisotropy decrease in TP1 (pretreatment) vs. controls. **(B)** Fractional anisotropy increases after bumetanide treatment. Significance maps (in red and yellow) overlaid on smooth anisotropy control image. The threshold was set at *P* < 0.05 (FEW Corrected) and minimum cluster size >5 voxels. Color scale represents *t*-values. FA, fractional anisotropy; TP, Time Point.

However, the whole brain analyses between pre- and post-treatment phases showed significant increases of FA particularly in the right hippocampus (*P* = 0.008), the cerebellum (*P* = 0.02) and in the left parahippocampal gyrus (*P* = 0.01) independent to the seizure frequency index after treatment (Figure 1).

### Bumetanide Effect on the Longitudinal Changes of White Matter

We hypothesized that bumetanide would have an influence on changes in DTI indices (FA and MD) of tracts. Based on the longitudinal analyzing of 18 tracts, significant reversal changes found in FA (FA increased) in the UF, ATR, and CST tracts over time that bumetanide administered. Additionally, MD decreased in the SLFt tract (Table 4). The observed changes in the FA and MD parameters following bumetanide administration were statistically significant in the right hemisphere. Based on the conventional MRI data, six of these patients had a lesion in the right side of the brain (data presented in Table 1). It should be noted that the remaining of the patients were MRI negative.

**Table 4:** The statistical significance of the time, hemisphere and response effects on FA and MD values in tracts of interest for mixed model analyzing regarding age, sex, TMI, and ICV as covariance.

**Location**		**Time**	**Response**	**Hemisphere**
		**F (*****P*****-value)**		
Forceps major	FA MD	0.942 (0.346) 0.79 (0.02)	0.051 (0.825) 0.8 (0.06)	– –
Forceps minor	FA MD	0.459 (0.507) 1.647 (0.202)	0.002 (0.962) 1.976 (0.173)	– –
SLFt	FA MD	0.212 (0.658) 12.456 (0.006)^**^	27.780 (0.0001)^***^ 1.012 (0.317)	0.239 (0.631) 0.290 (0.594)
SLFp	FA MD	0.502 (0.489) 3.508 (0.075)	1.786 (0.193) 0.149 (0.702)	61.639 (0.0001)^***^ 0.167 (0.685)
ILF	FA MD	0.023 (0.880) 0.323 (0.576)	3.265 (0.08) 2.166 (0.152)	7.251 (0.01)^*^ 14.998 (0.0001)^***^
ATR	FA MD	16.864 (0.0001)^***^ 2.181 (0.144)	6.214 (0.021)^*^ 2.172 (0.146)	2.872 (0.105) 5.993 (0.016)^**^
CCG	FA MD	0.618 (0.455) 0.042 (0.840)	6.690 (0.02)^*^ 2.625 (0.112)	0.282 (0.296) 0.008 (0.927)
CAB	FA MD	1.779 (0.194) 0.003 (0.959)	0.330 (569) 0.273 (0.607)	3.452 (0.075) 0.159 (0.695)
CST	FA MD	4.068 (0.056)^*^ 0.781 (0.383)	0.18 (0.678) 0.016 (0.901)	18.529 (0.0001)^***^ 5.489 (0.024)^*^
UF	FA MD	12.558 (0.012)^**^ 1.814 (0.182)	0.461 (0.502) 0.672 (0.417)	0.132 (0.720) 3.959 (0.051)^*^

* P < 0.05;

** P < 0.01;

*** P < 0.001. Bold values are statistically significant.

Moreover, our data showed that FA increased in the CCG, ATR, and SLFt which meaningfully correlated with the clinical response (Table 4). Data [Mean (SD)] of FA and MD in control group and pre- and post-bumetanide administration phases in patients [responders (*n* = 8) and non-responders (*n* = 4)] presented in Table 5.

**Table 5:** FA and MD values [Mean (SD)] in controls; Patients pre-, post- [responders (*n* = 8), and non-responders (*n* = 4)] 6 months bumetanide administration.

			**Control**	**Patients**			
				**Pre-treatment**		**Post-treatment**	
**Location**	**Side**				***P*-value**	**Responders**	**Non-responders**
Forceps major	FA MD e^−3^		0.58 (0.03) 0.79 (0.02)	0.59 (0.006) 0.8 (0.06)	0.2 0.22	0.56 (0.007) 0.8 (0.06)	0.56 (0.005) 0.8 (0.01)
Forceps minor	FA MD e^−3^		0.50 (0.03) 0.8 (0.04)	0.51 (0.003) 0.8 (0.03)	0.34 0.91	0.50 (0.04) 0.8 (0.04)	0.51 (0.003) 0.7 (0.02)
SLFt	FA MD e^−3^	L R L R	0.48 (0.03) 0.47 (0.03) 75 (0.04) 0.76 (0.03)	0.48 (0.02) 0.47 (0.05) 82 (0.03) 0.80 (0.03)	0.0027^*^ 0.47 0.001^*^ 0.42	0.48 (0.03) 0.48 (0.03) 0.8 (0.06) 0.8 (0.03)	0.45 (0.08) 0.47 (0.02) 0.8 (0.01) 0.7 (0.03)
SLFp	FA MD e^−3^	L R L R	0.47 (0.03) 0.45 (0.03) 0.75 (0.03) 0.75 (0.03)	0.48 (0.04) 0.44 (0.03) 0.79 (0.03) 0.79 (0.04)	0.54 0.04^*^ 0.15 0.94	0.48 (0.06) 0.46 (0.03) 0.8 (0.05) 0. 8 (0.08)	0.46 (0.02) 0.46 (0.02) 0.8 (0.04) 0. 7 (0.03)
ILF	FA MD e^−3^	L R L R	0.51 (0.05) 0.47 (0.04) 0.80 (0.04) 0.80 (0.03)	0.51 (0.05) 0.48 (0.04) 0.82 (0.02) 0.81 (0.04)	0.38 0.24 0.21 0.64	0.50 (0.05) 0.50 (0.05) 0.8 (0.04) 0.9 (0.01)	0.48 (0.05) 0.47 (0.03) 0.8 (0.02) 0.8 (0.03)
ATR	FA MD e^−3^	L R L R	0.45 (0.04) 0.47 (0.03) 0.74 (0.03) 0.76 (0.02)	0.45 (0.04) 0.45 (0.02) 0.77 (0.02) 0.79 (0.03)	0.15 0.001^*^ 0.69 0.2	0.49 (0.02) 0.47 (0.05) 0.77 (0.02) 0.78 (0.03)	0.47 (0.01) 0.46 (0.01) 0.77 (0.02) 0.77(0.03)
CCG	FA MD e^−3^	L R L R	0.55 (0.1) 0.52 (0.04) 0.75 (0.04) 0.74 (0.03)	0.54 (0.1) 0.53 (0.04) 0.79 (0.01) 0.79 (0.05)	0.0026^*^ 0.5 0.43 0.52	0.48 (0.06) 0.53 (0.02) 0.8 (0.01) 0.8 (0.01)	0.57 (0.01) 0.57 (0.01) 0.77 (0.04) 0.76 (0.05)
CAB	FA MD e^−3^	L R L R	0.38 (0.06) 0.41 (0.05) 0.82 (0.06) 0.82 (0.01)	0.39 (0.06) 0.40 (0.05) 0.87 (0.06) 0.91 (0.01)	0.95 0.13 0.51 0.36	0.39 (0.07) 0.42 (0.06) 0.88 (0.08) 0.93 (0.01)	0.38 (0.05) 0.42 (0.05) 0.9 0(0.01) 0.84 (0.07)
CST	FA MD e^−3^	L R L R	0.59 (0.02) 0.57 (0.03) 0.71 (0.02) 0.72 (0.02)	0.58 (0.02) 0.56 (0.03) 0.75 (0.02) 0.77 (0.02)	0.36 0.49 0.9 0.78	0.60 (0.02) 0.57 (0.02) 0.75 (0.04) 0.82 (0.01)	0.60 (0.02) 0.58 (0.04) 0.84(0.02) 0.71 (0.07)
UF	FA MD e^−3^	L R L R	0.45 (0.02) 0.44 (0.03) 0.79 (0.02) 0.80 (0.04)	0.45 (0.03) 0.43 (0.03) 0.82 (0.03) 0.84 (0.03)	0.84 0.0025^*^ 0.8 0.0022^*^	0.46 (0.05) 0.45 (0.04) 0.81 (0.05) 0.83 (0.04)	0.44 (0.01) 0.45 (0.02) 0.82 (0.04) 0.82 (0.04)

L, Left; R, Right.

* *P-value corrected for multiple comparison of 18 tracts (P = 0.0028)*.

### Bumetanide Effect on the Longitudinal Changes of Cognitive Functions

Cognitive measures of the control group, pre- and post-bumetanide administration phases can be seen in Table 6. The reaction time of patients in neutral, congruent, incongruent conditions, and also the error rates in the Stroop test were significantly higher in the patients compared to the healthy subjects. Increased reaction time, error percent and low performance rate in spatial memory test detected for patients relative to the control group. In addition, the higher reaction time in the N-back test observed in patient group. Following the 6 months bumetanide administration, percentage of errors in the spatial memory test reduced (*p*-value:0.001) and performance in the spatial memory enhanced (*p*-value: 0.001) after regarding age and sex as covariates; but no significant changes observed in association with the clinical response. These data suggest that bumetanide affect the memory function independent of the seizure frequency fluctuations.

**Table 6:** Cognitive test scores [Mean (SD)] in controls, Patients pre and post [responders (n = 8) and non-responders (n = 4)] 6 months Bumetanide administration.

	**Control**	**Patient**			
		**Pre-treatment**		**Post-treatment**	
			***P*-value**	**Responders**	**Non-** **responders**
**STROOP**					
Neutral	1.02 (0.2)	3.12 (2.3)	0.006^**^	1.32 (0.54)	3.25 (2.39)
Congruent	0.86 (0.1)	1.9 (1.6)	0.03^*^	1.16 (0.27)	2.85 (1.99)
Incongruent	1.16 (0.4)	2.23 (1.18)	0.008^**^	1.22 (1.22)	4.96 (4.21)
Error (%)	4.04 (0.8)	12.81 (14.78)	0.05^*^	2.01 (1.39)	7.66 (5.53)
**MEMORY**					
Mean Reaction Time	2.83 (0.6)	6.09 (2.93)	0.001^***^	4.38 (0.8)	6.23 (1.67)
Error (%)	20.9 (9.5)	33.90 (19.5)	0.05^*^	3.93 (6.62)	11.73 (11.05)
Performance (%)	79 (9.5)	66.09 (19.5)	0.05^*^	96.06 (6.62)	85.77 (12.73)
**N-Back**					
Error (%)	9.79 (8. 3)	20.07 (24.83)	0.18	12.43 (12.80)	34.08 (35.10)
Mean Reaction Time	1.15 (0.3)	1.15 (0.3)	0.03^*^	0.78 (0.1)	0.90 (0.1)
Performance (%)	90.9 (8. 3)	79.92 (24.83)	0.18	86.31(14.37)	63.64 (33.19)

* P < 0.05;

** P < 0.01;

*** P < 0.001.

### Associations Between Cognitive Functions and DTI Changes

**Figure 2:**
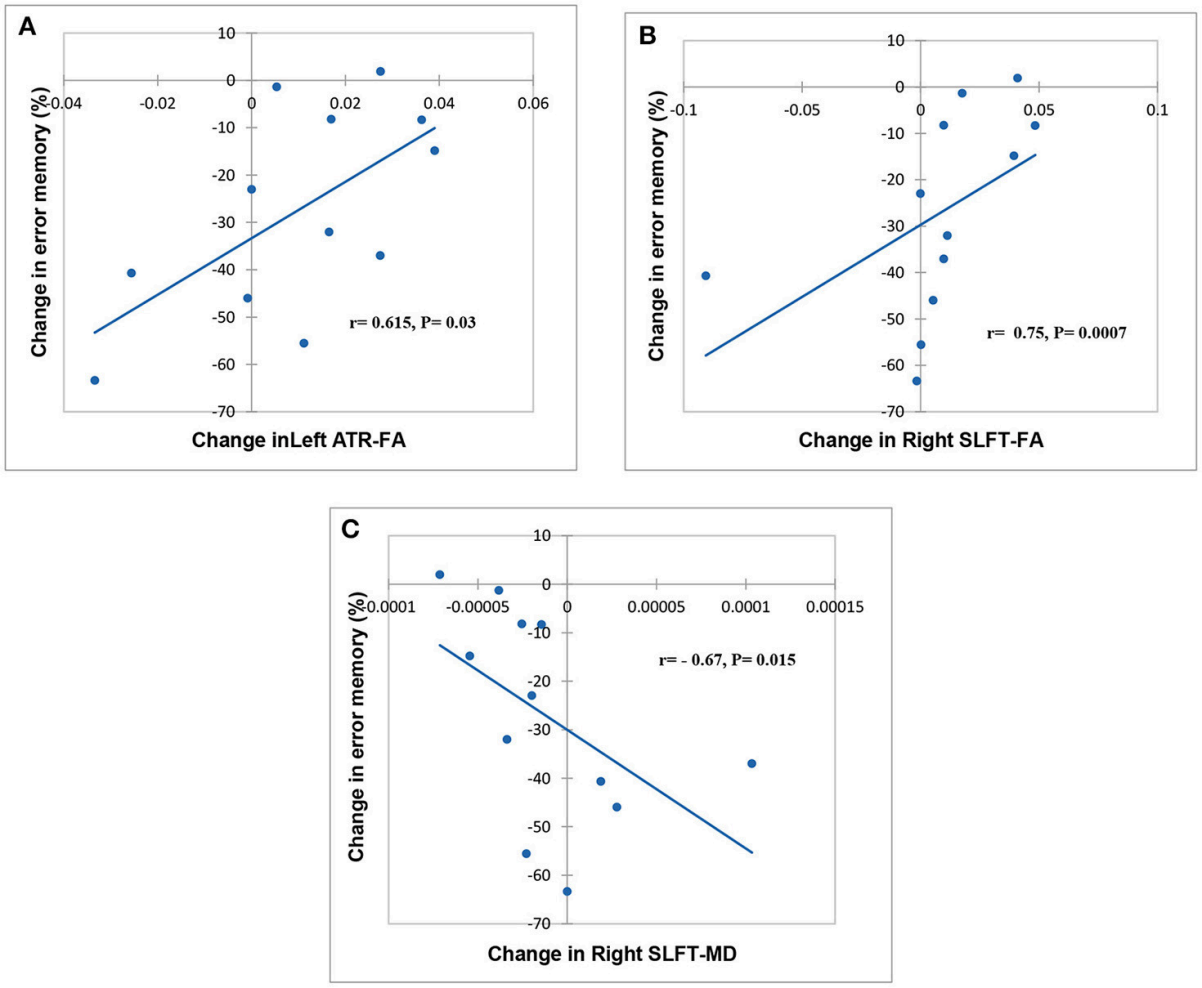
Scatter plot showing a relationship between change related to baseline in FA, MD, and cognitive task. Significant relationships between FA in left ATR, FA, and MD in the right SLFt and % error memory indicating, respectively in **(A–C)**.

Spearman correlation analysis of cognitive functioning and DTI changes revealed a significant relationship between changes in FA, MD, and cognitive indices. Statistically, are markable association observed between FA in left ATR, MD in the right SLFt, and the percentage of the errors in the spatial memory test (Figure 2).

## Discussion

In this study, we explored the association between brain microstructural abnormalities and different dimensions of the neurocognitive functioning after bumetanide administration in medication-resistant epilepsy. Significant changes in DTI metrics were revealed in different neuronal tracts in patients compared with control subjects and also in pre- and post-treatment phases of the patient group within a 6 month period. Moreover, this analysis successfully provided evidence for the existence of the hypothesized causal link between the indices of cognitive functions including reaction time and errors, and FA or MD values.

Currently provided data showed 67% responsiveness to bumetanide as an add-on therapy. Despite the low concentrations of the bumetanide in the brain following systemic administration (37, 38), recent studies indicated that bumetanide is able to control some aspects of neurological and neuropsychological disorders such as epilepsy (13, 20), schizophrenia (39, 40), Autism (41), and Parkinson disease (42). Previous researches have consistently documented a significant decline in the intracellular concentration of chloride and seizure frequency following the NKCC1 blockade by bumetanide (43–45). We also recently found that bumetanide could reduce the interictal spikes beside seizure frequency in medically-resistant epileptic patients (13). Nevertheless, the exact mechanisms by which bumetanide could control seizure and improve other chronic neurological symptoms are fairly unclear.

Although the combination therapy makes the accurate pharmacological interpretations difficult, it might be noticeable that 10 patients had been receiving different dosages of valproate within their background anticonvulsant regimen which signify the possible positive interactions. Valproate is a broad spectrum anticonvulsant drug that exerts its effects through several ways that could potentially underlie the observed clinical responses and cognitive impacts (46). Besides bearing significant molecular resemblance to GABA, valproate blocks both reuptake and catabolism of GABA (47) that possibly in concert with facilitation properties of bumetanide in restoring the functional polarity of GABAA receptor, increases the overall inhibitory operations of the brain. GABA neurogenesis (48) is another evident mechanism through which valproate might shift the balance toward more inhibition in long term perspective; the conditions that possibly in concert with facilitation properties of bumetanide in restoring the functional polarity of GABAA receptor, increases the overall inhibitory operations of the brain. Moreover, there are considerable pieces of evidence of neurogenesis which is induced by alterations in GABA homeostasis (49). Therefore, additive neurogenesis could be underway specifically when it comes to longer duration of administration.

Six patients had brain lesions while remaining of the cases were MRI negative. There have been works pointing to the increased expression levels of bumetanide-sensitive co-transporter, NKCC1, in hypoxic, edematous or secondary progressive hemorrhagic states in animal models of traumatic brain injuries (50, 51). On the other side, neuroimaging analyses have evidently shown that edema is a predominant component in epileptogenesis activities (52, 53). It might be deduced that bumetanide predominantly affects damaged brain microstructures due to the possible increased levels of expression within these affected areas.

Upgrading the observed correlation between increased FA in the CCG, ATR, and SLFt and better bumetanide response, to a more precise cause and effect model requires a well-controlled study that enables statistical inferences. However, it might be assumed that efficient neuronal connections shift the overall states of the brain toward more stable states and subsequent cognitive and clinical improvements.

In order to provide a framework for understanding the central effects of bumetanide in temporal lobe epilepsy, we investigated microstructural changes in the cerebral white matter. The possible mechanisms underlying TLE include disturbed myelination patterns and alteration of neuronal density (54, 55). After the implementation of the treatment protocol, the white matter voxel-based analysis demonstrated higher FA in the right hippocampus, medial temporal lobe, cerebellum and lower MD in the right occipital lobe and cerebellum. However, the analysis of eighteen tracts showed FA rise in the ATR, SLFt, and CCG. These changes associated with clinical improvement in response to bumetanide, and might reflect the structural reorganization. Our results are in line with previous reports (56, 57). Some reports indicated an acute reduction in MD without changes in FA after the seizure, which is different from the baseline (58). Moreover, studies on post-epilepsy surgery indicated plasticity in distinct areas which is related to microstructural reconstruction (58). Based on present findings, bumetanide could act on the central nervous system through inducing changes in the brain microstructure. According to the functional and structural imaging findings and newer definitions, epilepsy is a disorder of network (59). Presence of different probable patterns of axonal connectivity that could be altered in various disease states necessitates development of novel tools for analysis (60). Revealing quantitative aspects of neuroanatomical structures and associated pathological sequels or cognitive deficits, put the tractography approaches superior to the conventional analysis of MRI findings. The probabilistic nature of TRACULA is also a remarkable side since this property resolves the crossing fibers voxels problems and properly take the uncertainty component into account (61).

The decline in white matter integrity has been represented by the two key changes: the reduction in FA and enhancement in MD (62). One recently published study in schizophrenia reported the significant predictive role of the microstructural changes for cognitive performance such as attention and executive functions following treatment (63). Our results indicate white matter microstructural changes in SLFt, ATR, and CCG in responder patients. Spearman correlation analysis of cognitive functions and these brain changes indicated the significant relationships between longitudinal MD and FA values and error reduction in the spatial memory test. This would be probable since The SLFt tract connects the frontal, occipital, parietal, and temporal lobes and plays a fundamental role in the memory network (64, 65). The anterior thalamic nuclei (ATN) and their interconnecting fibers are important components of an extended hippocampal circuit for episodic memory (66). It seems that these essential tracts which passing through the temporal lobe and are affected in epileptic syndromes, mainly mediate cognitive processing, and consequently are responsible for the observed dysfunctions. It is highly possible that the bumetanide is exerting its effects through these pathways. While there have been some brain regions that are hallmarks for specific cognitive functions, regarding dynamic nature of the epilepsy which might alter the areas and their underlying connections during the course of the disease, neuroimaging analyses applied for the whole brain to detect potential contributing regions.

In addition to the research-based applications of probabilistic tractography approaches (67), recent pre-surgical assessments are benefiting from progress in neuroimaging analysis. TRACULA is increasingly used by neurosurgeons for more precisely-planned operations (61). However, to the best of our knowledge, this is the first study that has executed a TRACULA-based follow up analysis of one specific medication.

Previous studies indicated that FA value could be an appropriate representative of the white matter constitution (63). We showed that the integrity of white matter in CST and UF altered by bumetanide; that was independent of seizure frequency. However, small sample size and uncontrolled design are the major limitations of the current study, which warrant caution in interpreting the findings.

Finally, one of the mortality related phenomenon of epilepsy is the sudden unexpected death in epilepsy or “SUDEP” that frequently occurs in resistant states. The underlying processes of SUDEP are yet to be clarified but some cardiovascular related mechanisms such as the myocardial atrophy and leucocyte infiltration—due to the catecholamine surge of seizures- have been evidenced through microscopic analyses (68). Due to the possible cardiovascular contribution in SUDEP, drugs that cover both anticonvulsant and cardiac effects may be better candidate AEDs.

## Conclusion

In accordance with our findings, there is a wide microstructural distortion in the brain of epileptic patients which might be the cause of the observed abnormalities in associative behavior and cognitive impairments. Adjunctive bumetanide treatment may lead to the microstructural reorganization that mostly affects the epileptic regions; The outcome that could be the underlying contributor for its cognition improving effects.

## Author Contributions

ZG designed the experiment, collected data, contributed to the analysis and interpretation of data, and wrote the initial draft of the manuscript. LS contributed to the analysis and writing the manuscript. MO has contributed to data analysis and interpretation. AY contributed to the analysis and interpretation of data. AT designed the experiment and collected data. ES revised the draft of the manuscript. MH was main responsible for hypothesis making, general guidance and project management. The final version of the manuscript was approved by all the authors.

### Conflict of Interest Statement

The authors declare that the research was conducted in the absence of any commercial or financial relationships that could be construed as a potential conflict of interest.
